# Incidental Trifid Median Nerve Unveiled in a Lactating Mother

**DOI:** 10.7759/cureus.5809

**Published:** 2019-09-30

**Authors:** Md. Abu B Siddiq, M Tanveer Hossain Parash

**Affiliations:** 1 Physical Medicine and Rehabilitation, Brahmanbaria Medical College, Brahmanbaria, BGD; 2 Biomedical Science and Therapeutics, Faculty of Medicine and Health Sciences, Universiti Malaysia Sabah, Kota Kinabalu, MYS

**Keywords:** trifid median nerve, carpal tunnel syndrome, lactating mother

## Abstract

Several anatomical variations concerning the median nerve have surfaced in the medical literature. Among them, bifid median nerve or median nerve bifurcation with or without persistent median artery has been widely reported. Sporadic case reports describe median nerve trifurcation (trifid median nerve) as well. In the present report, we describe carpal tunnel syndrome manifestations in association with trifid median nerve unveiled incidentally under high-frequency musculoskeletal ultrasonogram in a lactating mother-a first in the medical literature.

## Introduction

The median nerve is one of the most important branches of the brachial plexus and supplies the wrist and two-third radial aspect of the volar hand. Its sensory supply reaches the hand going over the flexor retinaculum, whereas motor division passes underneath the retinaculum, except in some anatomical variations [1]. More than four decades have already elapsed since Lanz first described four possible anatomical variations of the median nerve within the carpal tunnel: a) anomaly related to thenar branch course, b) accessory median nerve branch at the proximal tunnel, c) high division of the median nerve into two branches with or without median artery or an aberrant muscle in it, and d) accessory median nerve branch at the distal tunnel [2]. Among them, bifid median nerve (variant c) has widely been described with a reported incidence of 2.8% [3,4]. On the other hand, only a few case reports describe trifid median nerve including the very first report by Yalcin et al. in 2011 [5]. However, we are yet to estimate the incidence of the trifid median nerve. In the present write-up, we take the privilege of describing the trifid median nerve in a lactating mother with carpal tunnel syndrome (CTS) manifestations, depicted incidentally under musculoskeletal ultrasonogram (MSUS) (Samsung Accuavix 10, South Korea; 2010).

## Case presentation

A 30-year-old woman on postnatal care presented with the complaint of pain, tingling, numbness sensation according to the distribution of the left median nerve. The complaints were more while using hands, including caring for her baby and performing other usual daily chores. Sometimes the pain ascended retrograde in the left forearm, though she denied any radiating neck pain. Because of the nocturnal rise of the symptoms, the patient had disturbed quality of sleep. We examined the patient’s hand and wrist. Positive Tinel’s at the carpal tunnel, Phalen’s and reverse Phalen’s maneuvers at wrist favored a CTS diagnosis. There were no thenar and hypothenar muscles wasting. Her thyroid status was normal. She was non-diabetic and had no inflammatory joint disorders either.

**Figure 1:**
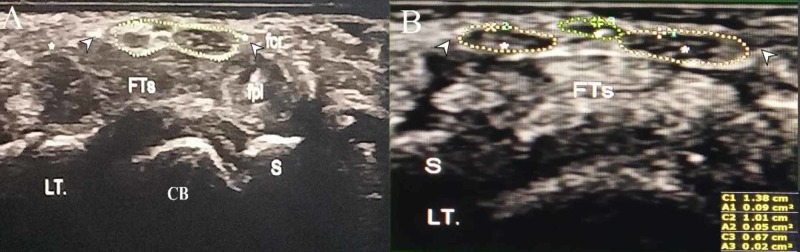
High-frequency linear transverse musculoskeletal ultrasonogram scan of left volar wrist. (A) transverse ultrasonogram of hypoechoic trifid median nerve segments encircled within hyperechoic sheath (dotted line). (B) trifid median nerve diameters (asterisks within dotted lines) for small, medium and large segments were 6.7 mm, 10.1 mm, and 13.8 mm, respectively. Arrowheads, flexor retinaculum; FTs, flexor tendons; CB, carpal bone; fcr, flexor carpi radialis tendon; fpl, flexor pollicis longus tendon; S, scaphoid; LT., left

Considering the above information, further scrutiny of the carpal tunnel under MSUS was planned. Oval-shaped, ovary-like median nerve morphology was found distorted, and the nerve was seen split into three divided hypoechoic segments, encircled within a hyperechoic sheath (Figures 1A, 1B). Diameters of the three divided median nerve segments were 6.7 mm, 10.1 mm, and 13.8 mm for small, medium, and large segments, respectively (Figure 1B). Divided nerve segments were found in close association, and there was no pulsating vessel among them (Figures 1A, 1B).

The condition was treated with diclofenac potassium (50 mg twice daily for three weeks) and the local application of ultrasound therapy over the flexor retinaculum for two weeks (0.8 watt/cm^2^/minute for 15 minutes). At her second visit, symptoms were found improved by approximately 50%. She was also advised how to adopt side-lying and foot-ball hold position while participating in baby care.

## Discussion

Lanz classified the anatomical variations of the median nerve in 1977 [2]. According to Lonie and colleagues, this classification criteria is not enough and should be revised to accommodate a trifid median nerve which could be done by modifying the criterion number three [3]. The trifid median nerve could also be identified incidentally in an asymptomatic individual [5].

Among anatomical variants, a bifid median nerve has widely been discussed as the most prevalent; however, this is in contrast with a recent cadaveric study performed in India [6]. In his study, Vashishtha reported that trifurcation of the median nerve was the most prevalent anatomical variant in the dissected human specimens [6]. Sometimes, both bifid- and trifid median nerve could be seen in the same patient as described by Yalcin et al., Duymus et al., and Rayegani et al. in a 54-year-old man, 46-year-old woman, and a 54-year-old woman, respectively (Table 1) [5,7,8].

**Table 1:** Research on trifurcation of median nerve MRI, Magnetic Resonance Imaging; MSUS, Musculoskeletal ultrasonogram

Research	Demography	Type of study	Co-morbidity	Diagnosis
Lonie et al. 2016 [3]	32-year-old man, trifid right median nerve	Case report	None	By clinical examination and during open release of carpal tunnel
Yalcin et al. 2011 [5]	54-year-old man, trifid median nerve in left side and bifid median nerve in right side	Letter to editor	None	Clinical examination and MSUS scanning (longitudinal and axial scans)
Vashishtha 2011 [6]	35 / 50 hands of dissected human bodies had median nerve anomalies	Descriptive study (cadaveric study); median nerve trifurcation has revealed in 23 (46%) hands	Not applicable	Dissecting hand of deceased human bodies
Duymus et al. 2013 [7]	46-year-old woman, left median nerve was trifid and right one was bifid	Case report	None	Clinical examination, MRI and MSUS
Rayegani et al. 2015 [8]	A 54-year-old woman, right median nerve was trifid and left median nerve was bifid	Case report	None	Median nerve anomalies were revealed under MRI, MSUS and during open release of carpal tunnel

Alongside physical examination, radio-imaging such as MSUS, magnetic resonance imaging (MRI) and nerve conduction study (NCS) is useful in delineating CTS and adjacent nerve anomalies [7,8]. Surgical exploration of the anomalous median nerve at the volar wrist has both diagnostic and therapeutic value [7,8]. MSUS has higher sensitivity and almost equal specificity to MRI in delineating peripheral neuropathy, hence, we did not perform the latter approach [9]. The role of NCS regarding compressive neuropathy is also inconclusive, so we did not go through it either.

In the present study, high-frequency MSUS depicted three variably-sized hypoechoic median nerve masses encircled within a hyperechoic sheath. There was no persistent median artery among them. Sizes of the bifurcated nerves are mostly similar; however, their sizes could vary, and the same could occur for trifurcated median nerve as well. In their published works, Yalcin et al. and Rayegani et al. described similar ultrasonographic cross-sectional areas (CSA) for the trifurcated nerve portions at the carpal tunnel [5,8]. However, a study by Duymus et al. reported dissimilar widths of the trifurcated median nerve, which also echoed in our case [7]. Divided median nerve segments could lie in close proximity, or they may remain wide apart [7,8,10]. In the present study, we documented that all the divided median nerve fragments were in close association. 

Trifid median nerve has already been described in limited cases. Alongside some above mentioned clinical features, the present study further differs from the previous cases based on the breastfeeding status. Pregnancy and breast-feeding are associated with increased CTS prevalence [11,12]. The resultant clinical manifestations could be better explained by fluid retention, hormonal fluctuations, nerve hypersensitivity, glucose level fluctuation, etc. [11,12]. As the individual in the present study was already on postnatal care, the fluid retention hypothesis cannot fully explain the CTS manifestations. But flexor tendon sheath injury due to repetitive use of hand during baby-care probably contributed to the pathology. As enlarged anomalous median nerve volume already compromised the carpal tunnel, fluid retention, and flexor tendon sheath thickening would have further compromised carpal tunnel and nerve vicinity. Additional aberrant vascular branch compressing the median nerve could proliferate CTS features; however, aberrant vascular pathology was not seen in the present study.

Carpal tunnel syndrome in breastfeeding women can be treated using the following interventions - oral anti-inflammatory drugs, vitamin-B combination, diuretics, intralesional steroid injection, splinting, local ultrasound and laser therapy, however, sometimes, surgical exploration of the carpal tunnel is required to release the compressed median nerve [12]. Here we treated the condition with diclofenac potassium and the focal ultrasound therapy over the flexor retinaculum. Further training on how to adopt side-lying and foot-ball hold position during baby care was also provided and seemed to be effective [12].

## Conclusions

To sum up, trifurcation of the median nerve is a rare clinical scenario. As Lanz classification appears inappropriate in accommodating trifid median nerve, revising it or launching a new criterion to classify median nerve anomaly seems rational. To the best of our knowledge, this report is unique in describing carpal tunnel syndrome in association with the trifid median nerve in a lactating mother. When planning the treatment of carpal tunnel syndrome, the possibility of a trifid median nerve in the compromised carpal tunnel should be kept in mind. Since a single case report does not make everything crystal-clear about the disorder, extensive research on various aspects of the trifid median nerve is warranted.
